# High-Frequency Current Transformers Cascade for Power Electronics Measurements

**DOI:** 10.3390/s22155846

**Published:** 2022-08-04

**Authors:** Maciej Chojowski, Marcin Baszyński, Robert Sosnowski, Aleksander Dziadecki

**Affiliations:** Department of Power Electronics and Energy Control Systems, AGH University of Science and Technology, 30059 Krakow, Poland

**Keywords:** current transformer, high frequency current measurement, bandwidth analysis

## Abstract

High-frequency current transformers (HFCT) are widely used to measure fast transient current. Their advantages are simple structure and relatively moderate price. Their lower and upper bandwidth are limited, but the HFCT can be easily applied to many measuring applications in power electronics. The disadvantages of HFCT are substantial dimensions and a large weight. The paper proposes a system of a cascade connection of two transformers, which allows us to reduce these disadvantages. The properties of such an HFCT combination were investigated and described. In the article, the expression for double current transformer transmittances is derived. The frequency response of the sensor was determined, and the results were verified in a practical arrangement. An experimental setup of a cascade CT connection was made and tested, allowing for fast-changing signals in transients to be measured. This paper presents the theoretical basis and results of laboratory work on a wide range of static and dynamic tests of the proposed sensor.

## 1. Introduction

Precise current measurement in power electronic systems is necessary for the operation of the closed control system and for the implementation of calculation algorithms [1,2]. Resistance current sensors with high band up to 2 GHz [1,2,3,4,5] are called current shunt resistors (CSR). The disadvantage of such resistors is the introduction of additional elements into the electronic circuit, which changes the topology of the system and introduces additional losses [1,2]. The CSR can also be designed for high currents [5]. The CSR without additional electronics (e.g., isolated amplifiers) does not ensure galvanic isolation of the measuring circuit from the supply circuit, which is considered as disadvantage. It is possible to add an isolated amplifier, but their bandwidth usually does not exceed 1 MHz; therefore, the application of CSR sensors is limited in practical power systems [2].

Hall sensors [1] are other current sensors that transfer both the DC and the variable component. The advantage of the sensor is the complete isolation of the measurement, and its disadvantage is a relatively low-frequency response. References [6,7,8,9] present a method of increasing the upper limit frequency of such sensors by adding an additional current sensor to the Hall sensor. An additional sensor can be a current transformer or a Rogowski coil. The Rogowski coil allows for the measurement of high currents because it has no core (no saturation). The advantage is the small size of the measuring coil [1] and the possibility to design the coil in the form of a printed circuit [10]. Its disadvantages include the relatively low-frequency response for high frequencies [11].

High-frequency current transformers (HFCT) [1,2,12,13] are characterized by high bandwidth and the possibility of measuring isolation. The current transformer in the low-frequency range is a high-pass filter [12,13], which eliminates this type of sensor in many power electronics applications [2,3,14,15]. Both CSR and HFCT allow them to be used to measure currents in power electronic systems, for example to determine the power of switching losses [2,3]. In order for the current transformer to be used, for example, to measure the impulse current of the transistor (which allows one to omit the constant and low-frequency components), it is necessary at the design stage to ensure that the lower limit frequency is as low as possible [3,12,16]. Another aspect is maximization of the upper cut-off frequency. In [12,14], it was determined that the parasitic capacitance of the winding with a measuring resistor has a large influence on the upper frequency value, but the inductance of the leads was omitted. In [16], the inductance of the leads was taken into account, which significantly influenced the frequency response of the transformer, even for a small value of the inductance of the leads. Based on the publications [12,14,15,16], it can be assumed that a necessary aspect to increase the transformer bandwidth is the minimization of the parasitic capacitance. The turn-to-turn capacity has a major impact on the value of the entire parasitic capacitance [17,18]. Reference [17] describes the effect of winding capacity depending on the number of turns, but also on the distance between turns. The influence of the core capacitance on the equivalent parasitic capacity was determined in [18], where the value of the turn-to-turn capacity and the scaling factor defining the influence of the core and the number of turns were determined.

Commercially available isolated current sensors provide precise current measurement, but their dimensions do not allow for non-invasive applications in the power circuit (e.g., measurement of the drain current of the transistor in the TO-247 housing). Only the ultramini series Rogowski coil allows for easy connection to the circuit, but it has a relatively low bandwidth (for power measurement) of only 30 MHz [19]. The Tektronix sensor has a large bandwidth (120 MHz), but its price and volume are a big disadvantage [20]. Commercial current transformers are also available, for example by Pearson, with a band up to 250 MHz [21]. Unfortunately, the dimensions are not comparable to small Rogowski coil.

The proposal is to develop a small, insulated sensor, with dimensions similar to the Rogowski coil, but with a larger bandwidth. For this purpose, a transformer with small dimensions can be used. A similar solution was presented in [3,16,22], where the first transformer was used, adjusted to the dimensions of the current measured, and an additional commercial sensor was added to measure the secondary current of the transformer.

The article is divided into five parts. Section 2 describes the overall problem and the proposed solution. Section 3 contains the theoretical analysis of the proposed measurement system. Section 4 is devoted to the practical implementation of the two HFCTs and the frequency response of the sensor. The article ends with a major summary and conclusions in Section 5.

## 2. Problem Analysis

### 2.1. Research Goal

**Figure 1:**
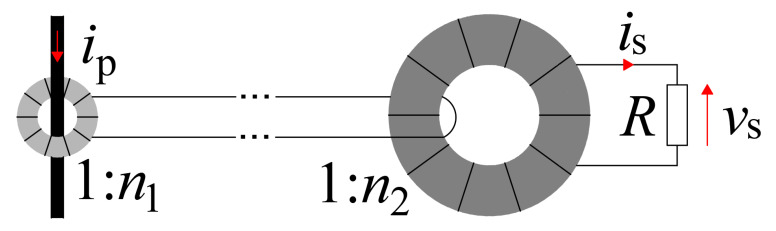
The concept of dual HFCTs and burden resistor *R*.

In the proposed work, the concept of a cascade connection of two current transformers was developed (Figure 1). The purposefulness and properties of such a combination have been demonstrated. The article describes the transformer model in the form of an operator transfer function. The analytical model for a system of two transformers is defined, and the advantages of the proposed cascade connection of current transformers ae indicated.

It was assumed that the core of the first transformer must have sufficiently small overall dimensions, enabling, for example, the current measurement directly on the transistor’s package (of typical sizes, e.g., TO-247). The small dimensions of the transformer core make it impossible to use a large number of turns of the secondary winding. Distances between individual turns must be large enough to limit the value of interturn capacities [17,18]. This is to obtain a sufficiently high value of the upper limit frequency. In the case under consideration, the number of turns of the first transformer was assumed to be *n*_1_ = 10, which seems to be the highest possible number of turns.

### 2.2. Benefits of the Proposed Connection 

The voltage generated on the resistor *R* should be sufficiently high due to the interference in the operating environment of the system. In the described case, it was assumed that the voltage should be *v_s_* = 100 mV for the maximum value of the measured current *i_p_*. The voltage across the resistor can be determined from the relationship:

(1) $$v_{s}=i_{p}\frac{R}{n}$$

Then, the burden resistance is:

(2) $$R=v_{s}\frac{n}{i_{p}}$$

The power dissipation of the burden resistor is given by:

(3) $$P_{R}=v_{s}\frac{i_{p}}{n}$$

Dependencies (1–3) are correct only when the frequency of the measured signal will be within the frequency range of the transformer.

The power in relationship (3) is inversely proportional to the converter ratio *n*. Therefore, to limit the power value (for a given current value), it is necessary to increase the system ratio. As previously explained for the first transformer, with the adopted design assumptions, it is not possible to increase the value of *n*_1_. The small dimensions of the transformer core also limit the energy stored in the electromagnetic field of the transformer, because at constant values of the magnetic induction and magnetic field strength (which can be assumed in the toroidal core), it is proportional to the core volume. Therefore, the power value of this transformer is limited. It is assumed that the power dissipated on resistance *R* has the largest share in the power of the transformer.

The application of the second HFCT with turn ratio *n*_2_ enables the solution of these problems, as a system is made with an overall ratio *n = n*_1_*∙n*_2_. The properties of such a measuring system are investigated in the further part of the article.

### 2.3. Related Work Comparison

The novelty of the proposed solution is the use of a two HFCTs, thanks to which it is possible to precisely measure current with galvanic isolation. In comparison to [3], the scope of the research is to make a small size current sensor to measure fast transient current (e.g., transistor drain current). 

The proposed silicon steel current transformer [3] can be adjust to specific package, but the results only show the general idea with measurements and do not provide an analytical approach. In [16], the switching current measurement probe is developed. The probe is combination of CT and commercial precision sensor. The results presented in [3,16] suggest the proposed solution to create an additional probe augmentation to commercially available sensor (e.g., Pearson Electronics, Palo Alto, CA, USA, CT or Tektronix TCP0030A). 

In [22], special attention is paid to dc component measurement and low-frequency ac signal saturation effects, with the aim of reducing distortion with low-frequency variation.

## 3. Bandwidth Analysis of the HFCT

### 3.1. General Model of a Single HFCT

**Figure 2:**
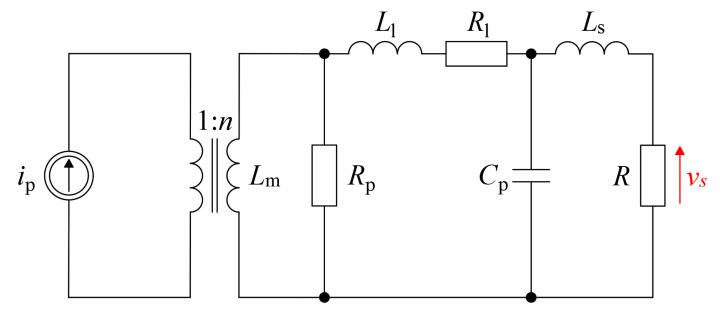
General model of HFCT.

The equivalent diagram of the current transformer was adopted as in Figure 2. It is an extension of the typical T equivalent diagram of the transformer. The equivalent circuit of the current transformer will be used in the following part to develop a model for low and high frequency.

The individual elements in Figure 2 represent:

*L_m_*—magnetizing inductance;

*R_p_*—core resistance;

*L*_l_—secondary leakage inductance;

*R*_l_—secondary winding resistance;

*C_p_*—parasitic capacitance; 

*L_s_*—lead inductance and inductance of the measuring resistor; 

*R*—burden resistor.

### 3.2. Low- and High-Frequency Response of the HFCT

**Figure 3:**
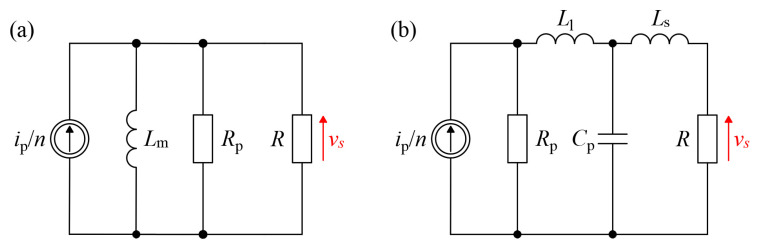
Proposed models of (**a**) low frequency and (**b**) high frequency.

To determine the transformer bandwidth, it is necessary to specify the transmittance description of HFCT and the analytical value of the cut-off frequency *f_L_* and the upper limit frequency *f_H_*. Both values will determine the band width (BW) and the range of currents measured by the HFCT. To define the model, it is necessary to formulate a replacement HFCT schematic for high and low frequencies. The proposed circuits are shown in Figure 3.

For low frequencies, we obtain the form transmittances:

(4) $$H_{L}\left(s\right)=n\frac{V_{s}\left(s\right)}{I_{p}\left(s\right)}=\frac{sL_{m}RR_{p}}{sL_{m}\left(R+R_{p}\right)+RR_{p}}=\frac{s\frac{RR_{p}}{R+R_{p}}}{s+\frac{1}{L_{m}}\frac{RR_{p}}{R+R_{p}}}$$

where:

(5) $$\omega_{L}=\frac{1}{\tau_{L}}=\frac{\frac{RR_{p}}{R+R_{p}}}{L_{m}}=\frac{R||R_{p}}{L_{m}}$$

The lower cut-off frequency of –3 dB of the current HF transformer is given by the following relationship:

(6) $$f_{L}=\frac{\omega_{L}}{2\pi }=\frac{R||R_{p}}{2\pi L_{m}}$$

As the core resistance is much greater than the measuring resistor (*R* ≪ *R_p_*), therefore:

(7) $$f_{L}\approx \frac{\omega_{L}}{2\pi }=\frac{R}{2\pi L_{m}}$$

A similar result was obtained in [12,14], but the influence of the lead resistance was also taken into account. The next step is to analyze the high-frequency model. The higher cut-off frequency *f_H_* depends on the influence of the leakage inductance *L_l_,* the core resistance, the parasitic capacitance *C_p_* and the lead inductance *L_s_*.

(8) $$\begin{array}{ll} H_{H}\left(s\right)=n\frac{V_{s}\left(s\right)}{I_{p}\left(s\right)} & =\frac{RR_{p}}{R_{p}+sL_{l}+\frac{sL_{s}+R}{s^{2}L_{s}C_{p}+RC_{p}s+1}}\frac{1}{s^{2}L_{s}C_{p}+RC_{p}s+1}\\ & =\frac{RR_{p}}{L_{s}L_{l}C_{p}}\frac{1}{s^{3}+s^{2}\left(\frac{R_{p}}{L_{l}}+\frac{R}{L_{s}}\right)+s\left(\frac{RR_{p}}{L_{s}L_{l}}+\frac{1}{L_{s}C_{p}}+\frac{1}{L_{l}C_{p}}\right)+\frac{1}{L_{s}C_{p}}\frac{R_{p}+R}{L_{l}}} \end{array}$$

**Figure 4:**
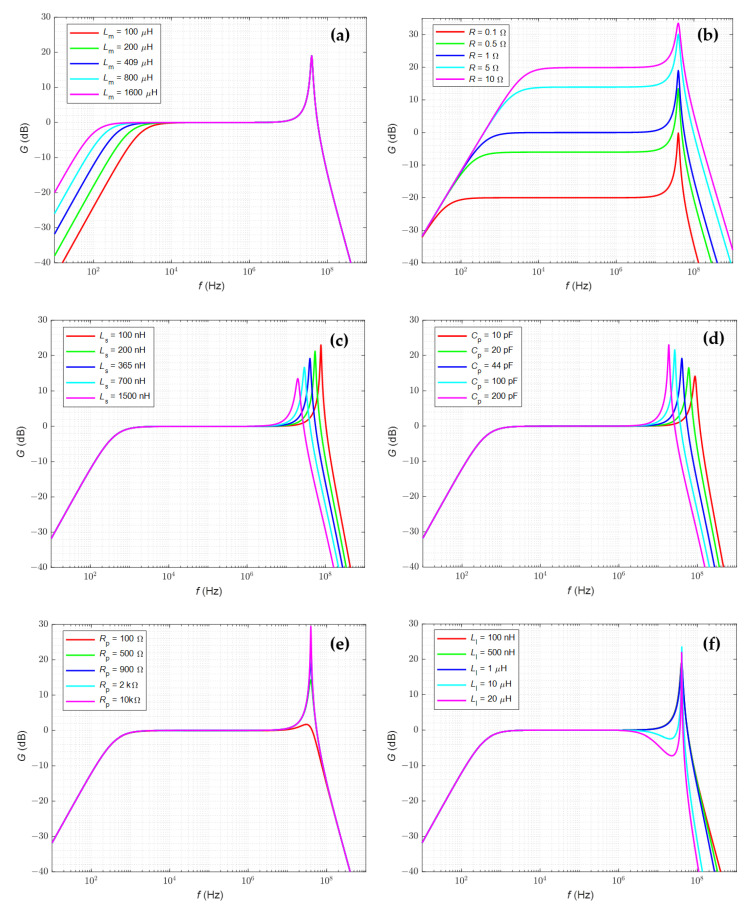
Bode plots of the HFCT model for variable values of the: (**a**) magnetizing inductance, (**b**) burden resistor, (**c**) lead inductance and inductance of the measuring resistor, (**d**) parasitic capacitance, (**e**) core resistance, (**f**) secondary leakage inductance.

Based on the transmittance of the transformer for low (4) and high (8) frequencies, the characteristics of the circuit gain *G* in function of frequency *f* (Figure 4) are developed. The influence of changes in the values of individual elements of the equivalent schemes on the obtained results is analyzed and presented. The initial values of the parameters are given in Table 1. 

The lower limit frequency is inversely proportional to the value of the magnetizing inductance *L_m_* (Figure 4a) and approximately directly proportional to the value of the burden resistance *R* (Figure 4b). A change in the *R* value also changes the gain of the system, and its impact on the resonant frequency is insignificant. The resonant frequency with which the upper cut-off frequency is associated increases with increasing inductance *L_S_* (Figure 4c) and decreases with increasing capacitance *C_p_* (Figure 4d).

As the core resistance *R_P_* increases (Figure 4e), the resonant gain value increases, but the resonant frequency remains approximately constant (low impact on the resonant frequency). Additionally, changes in the inductance *L_l_* over a wide range do not affect the bandwidth (Figure 4f). Only in the case of a relatively high *L_l_* value, a clear decrease in the gain value for frequencies lower than the resonance frequency is visible. Based on the results and analysis of the transmittance, it can be concluded that an important pole for the frequency response of transmittance (8) is:

(9) $$\omega_{H1}=\frac{1}{\tau_{LC}}=2\pi f_{H1}=\frac{1}{\sqrt{L_{s}C_{p}-{\left(\frac{R}{L_{s}}\right)}^{2}}\;\;}\approx \frac{1}{\sqrt{L_{s}C_{p}}\;\;}$$

Assuming a relatively large *L_l_* value and a small core resistance *R_p_*, the time constant may cause a decrease in the shape of frequency characteristic (Figure 4); then, the pole is important for the characteristic:

(10) $$\omega_{H2}=\frac{1}{\tau_{L}}=2\pi f_{H2}=\frac{R_{p}}{2\pi L_{l}}$$

The upper limit frequency can be determined from the following:

(11) $$f_{H}=\frac{1}{2\pi \sqrt{\frac{1}{f_{H1}^{2}}+\frac{1}{f_{H2}^{2}}}}$$

The bandwidth of the sensor is given by:

(12) $$BW_{-3db}=f_{H}-f_{L}$$

### 3.3. Model Analysis of Cascaded Connection of Two HFCTs

**Figure 5:**
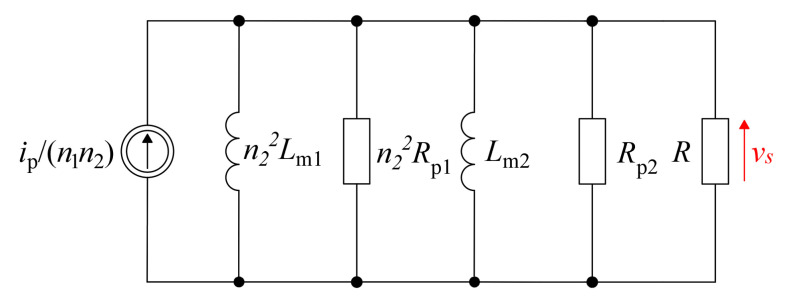
Proposed models of low-frequency cascaded connection of HFCTs.

The dependencies that determine the transmittance for a single HFCT allow for the definition of a simplified model for two cascaded connected transformers. The model for low frequencies is shown in Figure 5.

For low frequencies, the following transmittances are obtained:

(13) $$H_{2-L}\left(s\right)=\left(n_{1}n_{2}\right)\frac{V_{s}\left(s\right)}{I_{p}\left(s\right)}=\frac{s\frac{RR_{z}}{R_{z}+R}}{s+\frac{1}{L_{z}}\frac{RR_{z}}{R_{z}+R}}\;\;\;\;\mathrm{where}:\;R_{z}=\frac{n_{2}^{2}R_{p1}R_{p2}}{n_{2}^{2}R_{p1}+R_{p2}}\;\;\;\wedge \;\;\;L_{z}=\frac{n_{2}^{2}L_{m1}L_{m2}}{n_{2}^{2}L_{m1}+L_{m2}}$$

The lower limit frequency is defined by:

(14) $$f_{L}\approx \frac{R||R_{z}}{2\pi L_{Z}}$$

Based on the simulation results, it was found that the impact of the *R_P_* value on the frequency band is insignificant, so this element was omitted in the model for the cascade connection of two transformers. Furthermore, it was also assumed that the dispersion inductance *L*_1_ was relatively low, so its influence on the results obtained is small and can be neglected in further analysis.

**Figure 6:**
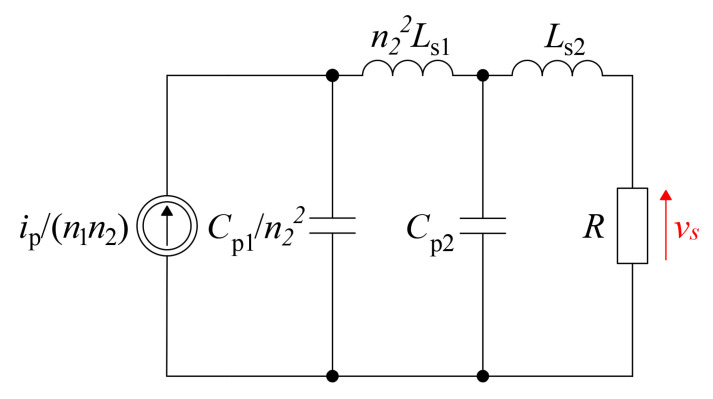
Proposed models of high frequency cascaded connection of HFCTs.

For high frequency (Figure 6), the overall transresistance is given by:

(15) $$H_{2-H}\left(s\right)=\frac{V_{s}\left(s\right)}{\frac{I_{p}\left(s\right)}{n_{1}n_{2}}}=\frac{R\frac{1}{L_{s1}C_{p1}}\frac{1}{L_{s2}C_{p2}}}{s^{4}+s^{3}\frac{R}{L_{s2}}+s^{2}\left(\frac{1}{L_{s2}C_{p2}}+\frac{1}{L_{s1}C_{p1}}+\frac{1}{n_{2}^{2}}\frac{1}{L_{s1}C_{p2}}\right)+s\left(\frac{1}{n_{2}^{2}}\frac{R}{L_{s1}}\frac{1}{L_{s2}C_{p2}}+\frac{R}{L_{s2}}\frac{1}{L_{s1}C_{p1}}\right)+\frac{1}{L_{s1}C_{p1}}\frac{1}{L_{s2}C_{p2}}}$$

Finally, the resonant frequency that defines the useful higher frequencies of the transformer can be defined as:

(16) $$f_{H0-1}=\frac{1}{\sqrt{L_{s1}C_{p1}}\;\;}\;\wedge \;f_{H0-c2}=\frac{1}{\sqrt{L_{s2}C_{p2}}\;\;}$$

The overall upper limit frequency can be determined from the dependence:

(17) $$f_{H0-2}=\frac{1}{2\pi \sqrt{\frac{1}{f_{H0-c1}^{2}}+\frac{1}{f_{H0-c2}^{2}}}}$$

## 4. Experimental Result 

### 4.1. Laboratory Setup 

**Figure 7:**
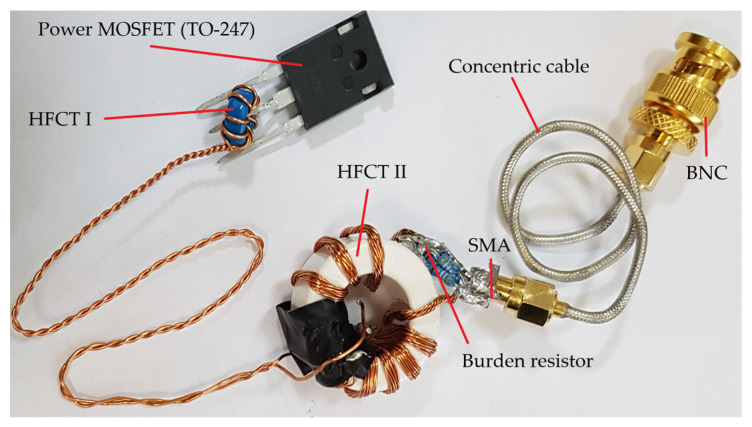
The proposed current sensors with dual connection of two HFCT—cascaded connection.

A practical sensor with cascade connected transformers based on the concept from Figure 1 is developed. The sensor system with an exemplary transistor (TO-247 package) is shown in Figure 7. Both transformers were made as toroids with MnZn as a core base material. The HFCT II was wired with litz wire to reduce the winding resistance. The HFCTs and their identified parameters are listed in Table 2 and Table 3.

### 4.2. Bandwidth of the Sensor

**Figure 8:**
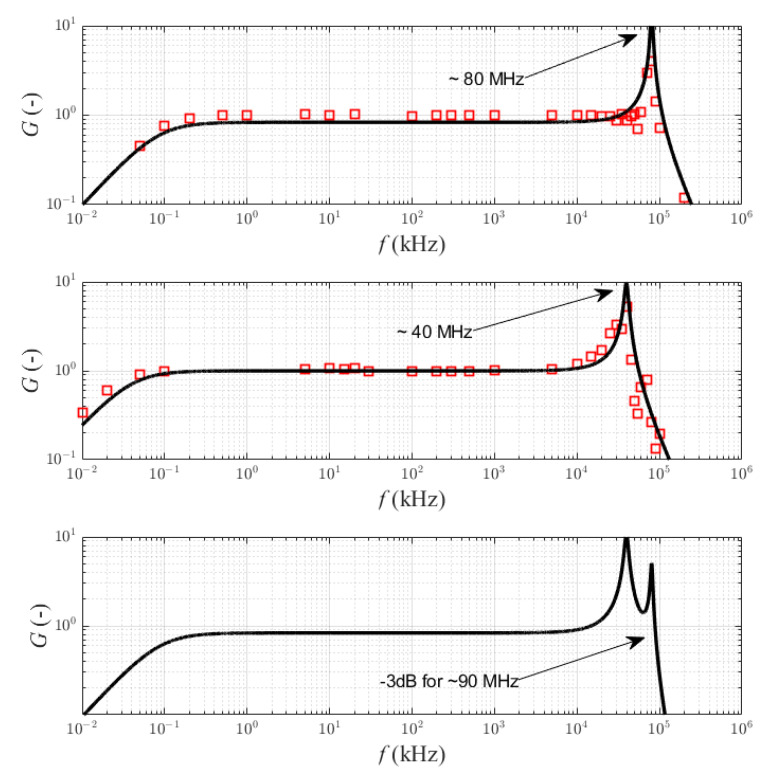
The Bode plots (*G*—gain vs. frequency) of the proposed current sensors with dual connection of two HFCTs, and the theoretical summaries of the frequency response for dual HFCT.

The gain characteristics in function of frequency (Figure 8) for both transformers were measured separately. Characteristics (measured points were marked as red symbol) were obtained by a high-frequency generator (with an output sinusoidal signal of about 100 mA). 

The continuous line marks the analytical characteristics of the proposed sensor obtained based on the relationships (4) and (8). No measuring points were defined for the cascade connection of the sensors, which was caused by the low value of the generator excitation signal. The characteristics for the transmittances (13) and (15) were plotted, and the values of the lower and upper frequencies were determined from the dependence:

(18) $$f_{L}=\frac{R||R_{z}}{2\pi L_{Z}}=86.5\;\mathrm{Hz}\;\;\;\wedge \;\;\;f_{H0}=\frac{1}{2\pi \sqrt{\frac{1}{f_{H0-c1}^{2}}+\frac{1}{f_{H0-c2}^{2}}}}=35.6\;\mathrm{MHz}$$

To verify the Bode plots, the −3 dB value for the cascaded HFCT sensor was confirmed in the next section based on the time response.

### 4.3. Time Domain Analysis

**Figure 9:**
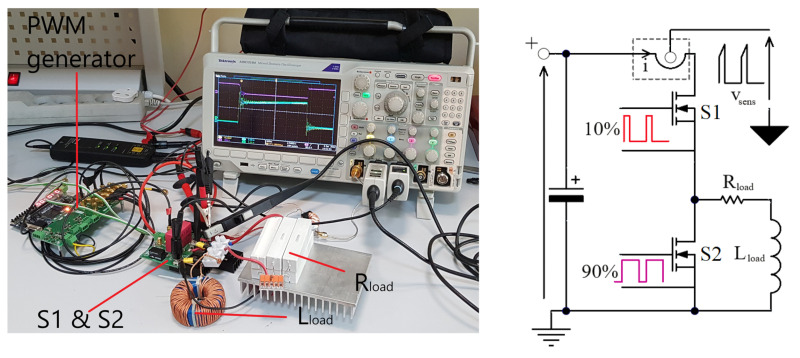
The laboratory setup for the pulse generation with a rapid current slope.

To obtain a dynamic response, the sensor was tested with a half-bridge circuit and a resistive-inductive load. Figure 9 shows the photography of the test circuit with a power circuit diagram. The capacitor (MKP 4.7 uF in parallel with electrolyte 100 uF) DC power supply was equal to 300 V. The resistance value was set to 2 Ω and the load inductor was 230 μH. The sensor was connected to the MDO3000 oscilloscope [23]. The power resistors and a choke were connected in series to a DC voltage source through a half-bridge system with SiC MOSFET transistors (CREE—C3M0060065D shipped by: Mouser Electronics, Mansfield, TX, USA). A gate source voltage was driven with a low duty cycle (about 10%) and fixed frequency *f* = 50 kHz. This arrangement made it possible to obtain a continuous current of the choke-resistor in the quasi-steady state. Consequently, the measured drain current (S1—FET) was a series of step pulses.

**Figure 10:**
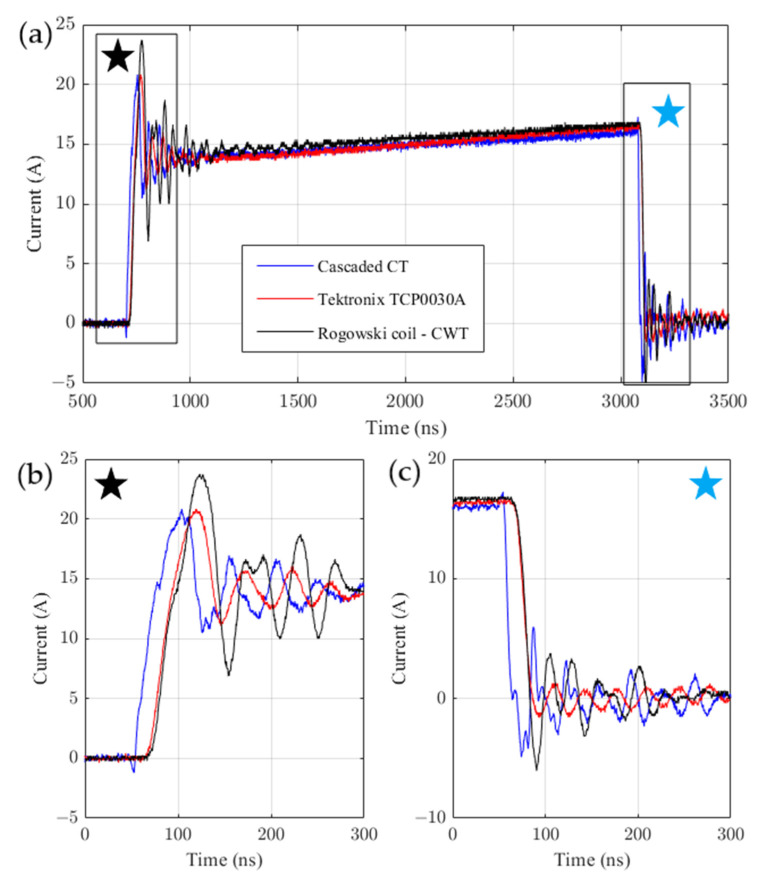
Response of the sensors for current pulse–transient response (**a**) of the transistor turn on (**b**) and turn off (**c**).

For verification, the Tektronix TCP0030A probe [20] with the 120 MHz band and the Rogowski CWT ultra mini coil with the 30 MHz band [19] were used. The measurement results for the dual HFCT probe, the TCP0030A probe and the Rogowski CWT coil are shown in Figure 10. 

The obtained results (Figure 10) of the time course analysis are presented in Table 4. The identified rise and fall slopes can be used to determine the sensor band. Most often, the rise and fall times are measured between 10% and 90% of the set value. The bandwidth was calculated based on the following equation [24]:

(19) $$BW_{-3\mathrm{db}}=\frac{K_{f}}{\min\left(t_{rise},t_{fall}\right)}$$

where the $K_{f}$ is factor value depends on the scope. Usually, the values differ from 0.35 to 0.45, but for high frequency, the correction factor can be higher [25]. For the –3 dB point as our definition of bandwidth for the oscilloscope, the relationship of rise time to bandwidth is a little different for a multi-pole response. The MDO3104 oscilloscope is rated for a 1-GHz bandwidth, and the shortest rise time is 400 ps [23]. The factor $K_{f}$ was assumed as $K_{f}$≈ 0.4.

Figure 10a presents measured waveforms. Figure 10b shows the close-up view of rising slopes, and Figure 10c shows the close-up view of falling slopes of these waveforms. Oscillations can be observed on all waveforms, but they are different from each other in the amplitude and phase. The impact of these differences can be significant, e.g., in case of the measurement of transistor switching power losses.

Both for the Tektronix probe and the Rogowski coil, the falling slopes are delayed by about 14 ns. Furthermore, the falling times are approximately twice greater than the falling time for CT. Based on the calculated value of the dual HFCTs’ sensor bandwidth, the calculated bandwidth is equal to 70.03 MHz. 

## 5. Conclusions and Future Works

The paper presents a concept of the current sensor, which was made as an series connection of two HFCTs. The current type output signals significantly reduce the noise impact to the measured current signal. The connection between both HFCTs can be extended even further, but the *L*_s_ will be increased and the bandwidth will be reduced.

The idea of two HFCTs can reduce the power dissipation of the burden resistor. This power is inversely proportional to the converter ratio *n* (Equation (3)). For the one small size HFCT used, we cannot achieve ratio *n* higher than approximately 10 for practical reasons. For nominal value vi and nominal current *i_p_,* the burden resistor power dissipation will be l/*n*∙*v_s_*. In the case of cascade system with the second HFCT ratio *n*_2_, the power dissipation will be *i_p_*/(*n*_1_*∙n*_2_) *v_s_*, which means *n*_2_ times lower—the dimensions of HFCT can be reduced.

General comparison with commercially available sensors and sensors from the references shows that the proposed sensor has comparable bandwidth. The bandwidth (−3 dB) of the sensor is close to 70 MHz, but for precision measurements, the resonant frequency should be $f_{H0-12}$ selected as the upper frequency boundary. The rise time of the inexpensive HFCTs can be significantly faster than the commercial TCP0030A. 

The proposed model of the HFCT differs from the commonly used model presented in [12] or [13]. The impact on the model response of the inductance *L*_s_ cannot be neglected, which was confirmed in the article. 

As the high-frequency current transformers can be made of various materials and in different construction ways, it is necessary to conduct optimization tests to construct a measuring system with the best possible parameters in the future.

## Figures and Tables

**Table 1:** Default parameters for frequency analysis.

Parameter	*L* *_m_*	*R*	*C_p_*	*L* *_s_*	*L* *_l_*	*R* *_p_*
Unit	μH	Ω	pF	nH	μH	Ω
Value	409	1	44	365	1	900

**Table 2:** Measured parameters of the HFCT I.

Parameter	*L* *_m_*	*R*	*C_p_*	*L* *_s_*	*L*_l_	*R* *_p_*	*n*	Outside Diameter	Internal Diameter	Length
Unit	μH	Ω	pF	nH	nH	Ω	-	mm	mm	mm
Value	409	866	44	365	100	-	1:10	10	6	4

**Table 3:** Measured parameters of the HFCT II.

Parameter	*L* *_m_*	*R*	*C_p_*	*L* *_s_*	*L*_l_	*R* *_p_*	*n*	Outside Diameter	Internal Diameter	Length
Unit	mH	kΩ	pF	nH	nH	Ω	-	mm	mm	mm
Value	1.9	2.47	87	45	7	1	1:12	27	20	17

**Table 4:** Response parameters of the tested sensor.

Parameters	*HFCTs*	*Tektronix*	*Rogowski coil*
Delay * [ns]	-	13.6	14.5
*t_fall_* [ns]	5.7	12.1	12.0
$BW_{-3\mathrm{db}}$ [MHz]	70.03	33.06	33.33

* Delay of the falling slope relative to HFCT’s.
